# *Salmonella* in meats, water, fruit and vegetables as disclosed from testing undertaken by Food Business Operators in Ireland from 2005 to 2009

**DOI:** 10.1186/2046-0481-65-17

**Published:** 2012-09-22

**Authors:** Sharon Duggan, Emily Jordan, Montserrat Gutierrez, Gaye Barrett, Tony O’Brien, Darren Hand, Kevin Kenny, June Fanning, Nola Leonard, John Egan

**Affiliations:** 1 National Reference Laboratory Salmonella, Department of Agriculture, Food and the Marine Laboratories, Backweston, Celbridge, Co, Kildare; 2 School of Agriculture, Food Science and Veterinary Medicine, University College Dublin, Dublin, Ireland

**Keywords:** *Salmonella*, Surveillance, Meats, Vegetables, Fruit, Food Business Operator, Ireland

## Abstract

**Abstract:**

Food Business Operators (FBO) are responsible for the safety of the food they produce and in Ireland those under the regulatory control of the Department of Agriculture, Food and Marine are required to provide summary data on microbiological tests undertaken as part of their food safety controls. These data are provided to the National Reference Laboratory through the 25 private laboratories undertaking the testing.

**Results:**

Over the five-year period *Salmonella* sp. was isolated from 0.7% of the 254,000 raw meat or raw meat products tested with the annual prevalence ranging from 0.5 to 1.1%. Poultry meats were consistently more contaminated than other meats with higher recovery rates in turkey (3.3%), duck (3.3%), and chicken (2.5%) compared with meats of porcine (1.6%), ovine (0.2%) and bovine origin (0.1%). *Salmonella* sp. was also isolated from 58 (0.06%) of the 96,115 cooked or partially cooked meat and meat products tested during the reporting period with the annual percentage positive samples ranging from 0.01 to 0.16%. A total of 50 different serotypes were recovered from raw meats over this period with the greatest diversity found in poultry samples (n = 36). Four serotypes, Kentucky, Typhimurium, Agona and Derby accounted for over 70% of all isolates detected on FBO testing over the period 2005 to 2009.

**Conclusions:**

Capturing microbiological data generated by Food Business Operators allows the regulatory sector access to a substantial amount of valuable data with the minimum financial outlay.

## Background

Foodborne disease is a widespread public health concern and although it’s global burden is unknown it is estimated to affect about 130 million Europeans every year [1,2]. *Salmonella* sp. are one of the most frequently reported causes of bacterial foodborne outbreaks in European Union Member States (EUMS) with 108,614 confirmed human cases in 2009 (23.7 cases per 100,000 population). Of these, 335 were confirmed in Ireland, corresponding to 7.5 cases per 100,000 of population [3]. In the EU, *Salmonella* Enteritidis and *Salmonella* Typhimurium are most frequently associated with human illness, with most *S.* Enteritidis cases associated with the consumption of contaminated eggs and poultry meat and most *S.* Typhimurium cases associated with the consumption of contaminated poultry, pork and beef products. Attribution studies show that pork is more frequently associated with human infections than beef [4]. While fruit and vegetables are sources of some human salmonellosis cases they are not regarded as contributing significantly to the burden of infection.

EU wide efforts to reduce food borne salmonellosis have targeted control measures at primary poultry production and on food safety criteria and food hygiene controls at abattoirs and food processing establishments. In Ireland, measures to control salmonellosis in poultry commenced in 1988 [5]. An EU-approved monitoring and control programme for *S*. Enteritidis and *S*. Typhimurium in breeding flocks of *Gallus gallus*, turkeys and ducks was later established under EU Council Directive 92/117/EEC. Subsequently, Directive 2003/99 and Regulation 2160/2003 introduced the requirement for *Salmonella* reduction targets in the various poultry production sectors. In addition, rules for sampling and testing certain food categories and the food processing environment were introduced under Regulation (EC) 2073/2005 and limits set for the presence of *Salmonella*. Although the application of EU wide controls for *Salmonella* in pig production have been under discussion for some years there is as yet no final agreement on how cost effective measures can be targeted [6]. In Ireland, a mandatory control programme for *Salmonella* in pigs was introduced in 2002 with limited effect but was revised in 2010 to ensure a more holistic application of controls along the production continuum.

As food is the most important vehicle for the transmission of *Salmonella* to humans it is desirable that that all data on contamination of the various food types are available for analysis. Official data on the microbiological contamination of food in Ireland are collected, evaluated and reported to the European Commission annually as required by Directive 2003/99/EC and published as part of the annual EU Summary Report on Trends and Sources of Zoonoses [3,7-10]. However food testing data generated by Food Business Operators (FBO’s), who undertake most of the microbiological testing and hold primary responsibility for food safety, are generally not collected or available for analysis. In Ireland, the importance of FBO data on *Salmonella* testing is recognised and systems are in place for its collection and analysis to allow more effective surveillance and the establishment of trends in pathogen incidence [1,11,12]. Commercial laboratories providing *Salmonella* testing for FBO’s regulated by the Department of Agriculture, Food and the Marine (DAFM) are required to submit information on this testing monthly to the National Reference Laboratory (NRL) *Salmonella*. Data on over 135,400 *Salmonella* tests conducted by FBO’s on raw and cooked meats from 2002 to 2004 have been published [12] and showed a downward trend in the recovery of this pathogen over that period. This communication presents the results on a further 457,569 tests undertaken by FBO’s on similar foods and vegetables over the 5-year period 2005 to 2009. The value of FBO test data on *Salmonella* in foods in Ireland is discussed as are trends and analysis of results.

## Materials and methods

Private laboratories, providing microbiological testing for FBO’s regulated by the DAFM in Ireland, are required to submit information on all *Salmonella* testing undertaken to the NRL on a monthly basis. In addition, all isolates must be submitted for full identification and the food or matrix from which they were isolated must be recorded. Isolates are serotyped at the NRL and all *S.* Typhimurium and *S* Enteritidis isolates forwarded to the Public Health Laboratory Service (PHLS), Colindale, UK or the Human *Salmonella* Reference Laboratory, National University of Ireland Galway, Galway, Ireland for phage typing.

A total of 25 laboratories provided isolates and information on their source over the period 2005 to 2009. The *Salmonella* isolation methods used in the laboratories are based on ISO 6579:2002 and tests are accreditated in most cases. These laboratories participated in regular proficiency tests including those organised by the NRL [13]. A template reporting form, outlining details of all testing undertaken on food samples, is issued to each laboratory to facilitate the uniform capture of data on a monthly basis.

Data in this report were selected in order to describe the occurrence of *Salmonella* in meats, water, fruit and vegetables in Ireland. The data provided did not allow determination of whether the products were domestically produced or imported. Some data may have been generated on foot of special investigations by FBO’s or as a follow up to food borne outbreaks and it was not possible to determine these except in the case of a *Salmonella* Agona outbteak during 2008.

All data are stored in a Microsoft Access based database (The FoodMicro Database) at the NRL. Data were collated and crude prevalence rates calculated for the 5-year period 2005 to 2009.

## Results

Bovine raw meat was the product most frequently tested over the 5-year period with a total of 149,761 tests reported followed by porcine meat (33,622 tests) and chicken meat (32,948 tests). Over the five-year period *Salmonella* sp. was isolated from 0.7% of the 254,000 raw meat or raw meat products tested with the annual prevalence ranging from 0.5 to 1.1%. Poultry meats were consistently more contaminated than other meats with higher recovery rates from meats of turkey (3.3%), duck (3.3%), and chicken (2.5%) compared with meats of porcine (1.6%), ovine (0.2%) and bovine origin (0.1%) (Table 1).

**Table 1:** **Crude prevalence rates for*Salmonella*sp. in raw meat and raw meat products, 2005 – 2009**

**Sample type**	** Number positive / Number tested (%)**				
	**2005**	**2006**	**2007**	**2008**	**2009**
Bovine	40/26977 (0.15)	47/33135 (0.14)	25/35134 (0.07)	55/26975 (0.2)	35/27540 (0.1)
Chicken	131/6836 (1.9)	76/8026 (0.9)	310/6027 (5.1)	238/6234 (3.8)	69/5825 (1.2)
Duck	0/131	2/108 (1.9)	9/142 (6.3)	6/59 (10.2)	0/68
Ovine	13/2773 (0.5)	2/2212 (0.09)	2/2183 (0.09)	1/2267 (0.04)	3/2195 (0.1)
Porcine	81/5962 (1.4)	94/6272 (1.5)	118/6649 (1.8)	142/5863 (2.4)	98/8876 (1.1)
Turkey	10/371 (2.7)	14/374 (3.7)	9/657 (1.4)	23/517 (4.4)	12/142 (8.5)
Edible fat/ dripping	0/775	3/525 (0.6)	2/625 (0.3)	1/772 (0.1)	0/506
Not specified	22/2380 (0.9)	29/4595 (0.6)	30/4579 (0.7)	40/4876 (0.8)	21/3837 (0.5)
**Total**	**297/46205 (0.6)**	**267/55247 (0.5)**	**505/55996 (0.9)**	**506/47563 (1.1)**	**238/48989 (0.5)**

**Figure 1:**
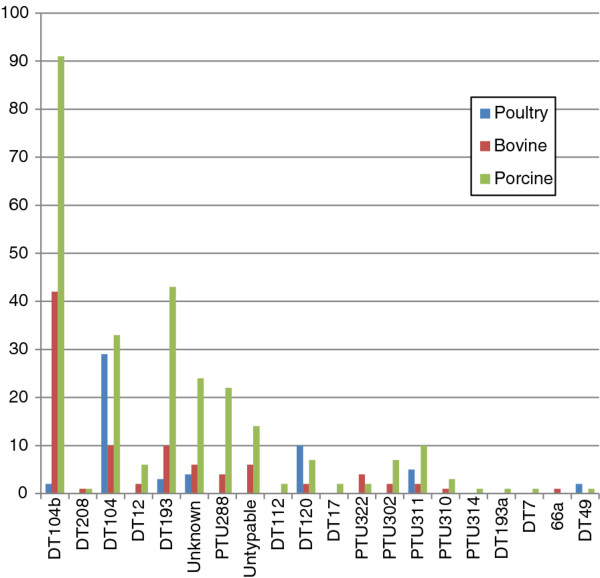
***Salmonella*Typhimurium phage types isolated from raw meat and raw meat products of porcine, bovine or poultry origin in the period 2005-2009.**

The *Salmonella* serotypes isolated from these samples are listed in Table 2. A total of 50 different serotypes were recovered from raw meats over this period. The greatest diversity in serotypes was found in poultry samples (n = 36) although a single serotype, S. Kentucky, accounted for 50% of all isolates. Of the 32 serotypes isolated from samples of porcine origin, *S*. Typhimurium and *S*. Derby were the most common, accounting for 272 (51%) and 92 (17%) of isolates, respectively. Fewer serotypes were recovered from samples of bovine origin (n = 21) with *S*. Typhimurium accounting for 46% of all isolates and predominating for all years, with the exception of 2007. *S*. Typhimurium phage type DT104b was the most frequently isolated phage type from bovine (45%) and porcine (34%) samples, while DT104 accounted for 53% of phage types recovered from poultry (Figure 1).

**Table 2:** **The number of*Salmonella*serovars isolated in Ireland from Poultry, Bovine and Porcine samples of raw meat and raw meat products, 2005–2009**

***Salmonella*Serotype**	**Poultry**					**Bovine**					**Porcine**				
	**2005**	**2006**	**2007**	**2008**	**2009**	**2005**	**2006**	**2007**	**2008**	**2009**	**2005**	**2006**	**2007**	**2008**	**2009**
*Salmonella* Agona	58	16	40	12	19	1	1								1
*Salmonella* Altona											1		2		
*Salmonella* Anatum		1	1		1							1			
*Salmonella* Bareilly			2												
*Salmonella* Bergen												1			
*Salmonella* Blockley		1			1										
*Salmonella* Braenderup															1
*Salmonella* Brandenburg			12	3			1	4	1				1		
*Salmonella* Bredeney	2	2		1			1	1	4	2	1	3	11	11	3
*Salmonella* Butantan													1		
*Salmonella* Cerro		2													
*Salmonella* Colindale															2
*Salmonella* Corvallis			1												
*Salmonella* Derby	2	3	1			4	3	3	7	2	18	15	20	19	20
*Salmonella* Dublin						8	11	6	6		1			1	
*Salmonella* Enteritidis	1	1	12	1	1			1					1	2	
*Salmonella* Goldcoast					1						1				
*Salmonella* Give														4	
*Salmonella* Hadar		6		2	1	1	1								
*Salmonella* Havana							1								
*Salmonella* Heidelberg	1	2											1		
*Salmonella* Indiana	3	1	1	7											
*Salmonella* Infantis	6	2	2	5		3		1		6	3		1	5	
*Salmonella* Kedougou											2				
*Salmonella* Kentucky	16	15	231	174	23	1		1	3		1	8	5		1
*Salmonella* Kibi		2													
*Salmonella* Kottbus		3	2	4								1			
*Salmonella* Lexington	1														
*Salmonella* Livingstone	7		1	1						1	1		1		
*Salmonella* London						1			1	1	3	3	4	15	8
*Salmonella* Mbandaka	8	16	2		2										
*Salmonella* Manhattan											6	2	1		
*Salmonella* Minnesota				17	2										
*Salmonella* Montevideo					1										
*Salmonella* Munchen				3											
*Salmonella* Newport			1	1	3										
*Salmonella* Nottingham				1			1		1						1
*Salmonella* Ohio				1				1							
*Salmonella* Orion		1	1		1		2								
*Salmonella* Panama							1						2		
*Salmonella* Paratyphi B / Java		4	5	13											
*Salmonella* Poona	2	1						1			3		1		
*Salmonella* Reading									1		1	5	2		
*Salmonella* Rissen								2	1					1	
*Salmonella* Saintpaul	2	1		1	4				1						1
*Salmonella* Schwarzengrund			3	1							1				
*Salmonella* Senftenberg	2										1				
*Salmonella* Thompson	1			1											
*Salmonella* Typhimurium	27	5	3	8	12	19	23	4	26	21	35	46	56	78	57
*Salmonella* Unnamed	2	7	2		3	2	1				2	9	1	2	3
*Salmonella* Virchow			2	2	2									1	
Presumptive			3	8	4				3	2			7	3	
**Total**	**141**	**92**	**328**	**267**	**81**	**40**	**47**	**25**	**55**	**35**	**81**	**94**	**118**	**142**	**98**

Excluding testing where the meat type was not specified, a total of 83,607 tests was undertaken on cooked or partially cooked meats compared with 230,530 tests on raw meat and raw meat product. Testing levels for all cooked or partially cooked meats were lower than for raw meats except for pig meat where 39,034 cooked or partially cooked products were tested compared with 33,622 on raw meat. *Salmonella* sp. was isolated from 58 (0.06%) of the 96,115 cooked or partially cooked meat and meat products tested during the reporting period with the annual percentage positive samples ranging from 0.01 to 0.16% (Table 3). In general, the annual recovery of *Salmonella* sp. from these products was low and results were distorted by 27 isolates of *S.* Agona from pork products associated with investigation of a foodborne outbreak in 2008. The 54 isolates recovered from specified cooked meat sources were identified as *S*. Agona (n = 37), *S*. Typhimurium (n = 6), *S*. Poona (n = 4), *S*. Derby (n = 2), *S*. Aberdeen (n = 1), *S*. Brandenberg (n = 1), *S*. Kentucky (n = 1) and *S*. Reading (n = 1) with one isolate unidentified. *Salmonella* serotypes were isolated on four occasions from ready-to-eat foods including fruit and vegetables.

**Table 3:** **Results of tests for*Salmonella*sp. from cooked meat/cooked meat products and ready-to-eat foods including vegetables, fruits and other samples, 2005–2009**

**Food type**		** Number positive / Number tested (%)**				
		**2005**	**2006**	**2007**	**2008**	**2009**
Cooked or partially cooked meat and meat products	Bovine	0/638	2/1808 (0.11)	0/1328	7/4794 (0.15)	0/6595
	Chicken	2/2296 (0.09)	2/3385 (0.06)	1/3371 (0.03)	2/6932 (0.03)	0/8373
	Duck	0	0/1	0/2	0/1	0/6
	Ovine	0/30	0/37	0/90	0/212	0/140
	Porcine	1/4529 (0.02)	0/5373	7/4068 (0.17)	27/10393 (0.27)	3/14671 (0.02)
	Turkey	0/682	0/694	0/575	0/1370	0/1213
	Not specified	0/2147	0/3541	2/3283 (0.06)	2/1430	0/2107
	Total	3/10322 (0.03)	4/14839 (0.03)	10/12717 (0.08)	38/25132 (0.16)	3/33105 (0.01)
Other foods/water	Ready-to-eat foods	0/3580	1/5452 (0.02)	1/8120 (0.01)	1/9393 (0.01)	0/10227
	Vegetable and fruit	0/3079	0/3490	1/3477 (0.03)	0/2517	0/1545
	Food grade water	0/752	0/520	3/375 (0.8)	0/416	0/305
	Other/not specified	3/10486 (0.3)	1/11604 (0.01)	4/11244 (0.04)	17/11735 (0.14)	9/9137 (0.10)
	Total	3/17897 (0.02)	2/21066 (0.01)	9/23216 (0.04)	18/24061(0.07)	9/21214 (0.04)

## Discussion

Data presented in this communication together with data from the 2002 – 2005 communication [13] show results of *Salmonella* tests on over 592,987 food samples in Ireland and provide the most comprehensive data set available for the period. In general, the downward trend in the recovery of this pathogen from raw Irish meats observed from 2002 to 2004 continued over the 5-year period reported here. The importance of poultry as the major source of foodborne salmonellosis is also reflected in the higher isolation rates of the organism from poultry meat in this study (2.5% positive samples compared with 0.39% for other meats). Data collected also show pig meat as a potential source of *Salmonella* sp. with *S*. Typhimurium and in particular DT104 and DT104b being the predominant phage types. The higher contamination rates recorded in raw meat in 2007 (0.9%) and 2008 (1.1%) were largely attributed to additional isolations from poultry meats associated with investigation of an *S*. Agona outbreak and on more targeted monitoring by the pig processing sector as part of the *Salmonella* control programme.

Results of FBO testing supplement the limited test data available from official testing during the period. For instance, official testing undertaken on porcine meats in 2008 show 0.3% of the 322 fresh meat samples tested were positive with none of the 28 samples testing positive in 2009. Limited official testing of broiler neck flap samples in 2009 showed 14% of the 250 tested at slaughter were positive for *Salmonella* with 2.6% of 116 samples collected at processing or cutting plants also positive [3]. No isolations were found on official testing of ready-to-eat meat products or vegetables in 2009. Although *Salmonella* was only isolated from one of the 14,108 samples of vegetables and fruit tested over the period reported here, the organism has been found occasionally on official testing of these foods in Ireland [3,7-10]. An overall *Salmonella* isolation rate of 0.6% was reported from these foods within EU in 2009 with only seven EUMS recording isolations [3].

Four *Salmonella* serotypes, Kentucky, Typhimurium, Agona and Derby accounted for over 70% of all isolates detected on FBO testing over the period 2005 to 2009. *S.* Kentucky and *S.* Agona were primarily associated with poultry meat, *S.* Derby primarily with pig meat and *S.* Typhimurium with all meat types though most often with pig meat. The prevalence of *S.* Kentucky increased dramatically in a number of broiler production companies in 2007 and 2008 but levels have been dropping in recent times (unpublished data). It is not uncommon for certain serotypes to become established in individual production companies as was the case with *Salmonella* Mbandaka which Gutierrez *et al*. [14] found to be established in one broiler production company in Ireland, possibly linked to a contaminated hatchery. *S.* Derby is widespread in the pig production sector in Ireland and the EU and was the third most frequent isolate from pig meat in the EU in 2009 [3]. Both *S.* Kentucky and *S.* Derby are rarely associated with human infection in Ireland [15] but accounted for 0.4% to 0.7% of human cases respectively in EUMS in 2009 [3]. *S.* Agona was associated with a cluster of human infections in Ireland in 2005 and subsequently with a larger European outbreak involving an Irish food company in 2008 [16].

While it is generally accepted that the true burden of human salmonellosis may be considerably larger than the reported incidence there is no doubt that the level of human infection is declining in most EUMS including Ireland [15]. The five-year EU-trend (2005 – 2009) shows a continuing decrease with 17.4% fewer cases in 2009 compared to 2008 [17]. While *S.* Enteritidis and *S.* Typhimurium are the predominant serotypes causing human illness in both the EU and Ireland, the decline in human salmonellosis cases is mainly attributed to the reduction of *S*. Enteritidis in eggs and flocks of laying hens, even though other control measures along the food chain may have contributed [3]. While the overall incidence of *S.* Typhimurium cases in humans in Ireland as in EUMS has remained stable its relative importance as a cause of infection has increased. The predominant phage types (DT104b, DT104 and DT193) found in humans infections were also those most frequently found in meats in this study. *S.* Typhimurium is predominant in the pig sector with two EU baseline studies showing a Community observed prevalence of *Salmonella*-positive pigs of 10.3% at slaughter and 30.9% of breeding pig holdings infected [18]. These data support the view that the introduction of effective *Salmonella* control programmes is important although consensus on EU-wide control measures has not yet been reached.

Not all salmonella infections are contracted locally or through consumption of local produce. It is estimated that in Ireland and the United Kingdom a ratio of 1:1 exists between domestic and imported cases. Travel associated cases in older people in Ireland were twice as likely to be due to *S.* Enteritidis than *S.* Typhimurium. In contrast, indigenous cases were twice as likely to be due to *S.* Typhimurium than *S.* Enteritidis [15], again suggesting that control of *Salmonella* in the pig sector is necessary. Although foods imported into Ireland and EU [19] have been contaminated with *Salmonella* there are no specific data highlighting any increased risks from imported products. A total of 50 different *Salmonella* serotypes were isolated from food products in this period compared to the 39 recorded by Jordan et al. [12] from 2002 to 2004, which may reflect to some extent the continuing globalisation of food production.

Data produced from testing undertaken by FBO’s are not generally collected and analysed centrally to enhance food safety controls in most EUMS or included in the analysis of trends and sources of zoonoses at EU level. This is in part due to concerns over the reliability of the data particularly in relation to correctly categorising the food products from which isolates are recovered. It is often difficult for laboratories to correctly classify some food samples, particularly differentiating between ready-to-eat / cooked or partially cooked submissions and this often leads to difficulty for the NRL categorising samples correctly. As reliable and accurate information on foodborne pathogens, particularly food-pathogen combinations is necessary [20], DAFM introduced additional measures in 2011 requiring FBO’s to more accurately categorise the food sample submitted for microbiological testing and this should facilitate better classification and identification of high risk products over time. In addition, all *Salmonella* isolates must be submitted to the NRL irrespective or whether or not a food product is placed on the market.

There continues to be some cross contamination of samples within laboratories as evidenced by a number of cases where the positive control organisms used for quality control are recovered from samples. For instance, the 12 isolations of *S.* Poona and *S.* Nottingham reported here from raw meat and products and the 4 isolations reported from cooked meat and products are most likely the result of such cross contamination. Previously, the NRL has reported on this issue [21,22] and DeLappe et al. [23] also reported on 23 incidents concerning 56 *Salmonella* isolates where cross contamination was the most likely source.

## Conclusions

Capturing microbiological data generated by FBO’s allows the regulatory sector access to a substantial amount of valuable data with the minimum financial outlay. As FBO data include a range of testing undertaken outside those required for mandatory EU reporting, it offers the potential for more detailed food safety trend analysis and when coupled with the requirement for all isolates to be sent to the NRL, more rapid alerts of food safety issues. While there is an ongoing need for FBO’s to ensure that HACCP controls are effective at all stages of the food chain, improvements in the microbiological quality of food may not result in an immediate corresponding reduction in human infections [24,25]. Lack of controls during food processing, in the food service industry and in the home was deemed to be a critical factor in failure to reduce human infections [26]. Ravishanker et al. [25] and Gormley et al. [27] estimated that approximately 25 – 60% of cases could be attributed to improper food handling practices in the home. In Ireland, the Food Safety Promotion Board is promoting a public awareness campaign on safe food handling practices in the home [28].

## Competing interests

None of the authors have any competing financial or other interests that could influence or bias the contents of this paper.

## Authors’ contributions

SD and EJ coordinated the collection of data and together with JE were the primary authors of the paper. GB, TO’B and DH collected data and typed isolates. JE, MG, KK, JF and NL acted as supervisors of the work and its quality control at various periods. SD’s current address is c/o British Antarctic Survey, Stanley, Falkland Islands. EJ’s current address is c/o Forsensic Science Laboratory, Dublin. All authors read and approved the final manuscript.
